# Non‐Stationary Outcome of Alternating Hemiplegia of Childhood into Adulthood

**DOI:** 10.1002/mdc3.13388

**Published:** 2021-12-29

**Authors:** Marco Perulli, Josephine Poole, Giulia Di Lazzaro, Sasha D'Ambrosio, Katri Silvennoinen, Sara Zagaglia, Diego Jiménez‐Jiménez, Domenica Battaglia, Sanjay M. Sisodiya, Simona Balestrini

**Affiliations:** ^1^ Child Neurology and Psychiatry Unit Fondazione Policlinico Universitario Agostino Gemelli IRCCS Rome Italy; ^2^ Department of Neuroscience Catholic University Of The Sacred Heart Rome Italy; ^3^ Department of Clinical and Experimental Epilepsy UCL Queen Square Institute of Neurology London United Kingdom; ^4^ Department of Systems Medicine Tor Vergata University Rome Italy; ^5^ Neurology Unit Fondazione Policlinico Universitario Agostino Gemelli IRCCS Rome Italy; ^6^ Dipartimento di Scienze Biomediche e Cliniche “L. Sacco” Università degli Studi di Milano Milan Italy; ^7^ Chalfont Centre for Epilepsy Bucks United Kingdom; ^8^ Neuro Center Kuopio University Hospital Kuopio Finland; ^9^ Neuroscience Department Meyer Children Hospital Florence Italy

**Keywords:** alternating hemiplegia of childhood, ATP1A3, adult, movement disorders, regression

## Abstract

**Background:**

Although described as non‐progressive, alternating hemiplegia of childhood (AHC) can display a sudden deterioration, anecdotally reported mainly in childhood. Outcome in adulthood is uncertain.

**Objectives:**

Aim of this study is to describe the long‐term follow‐up of neurological function in adults with AHC.

**Methods:**

Seven adults with AHC were included in this retrospective single‐center study. Clinical history and previous investigation data were gathered from the review of medical records. Video‐documented neurological examination was performed at the last follow‐up visit in four out of the seven reported indivisuals.

**Results:**

Over a median follow‐up of 16 years, neurological outcome and trajectories were heterogeneous. All individuals showed new neurological signs or symptoms. Three experienced a serious irreversible neurological deterioration after prolonged quadriplegic episodes and/or status epilepticus in their second or third decade. One patient died at age 29.

**Conclusions:**

This video‐series suggests that AHC in adulthood is not stationary; larger cohorts are needed to identify genotype–phenotype correlations and clinically useful outcome predictors.

Alternating hemiplegia of childhood (AHC) is a rare neurodevelopmental condition with onset before 18 months of age, characterized by recurrent attacks of hemiplegia involving one or both sides of the body that typically resolve on sleep, and other paroxysmal disturbances including epilepsy, paroxysmal dystonic attacks, oculomotor abnormalities, and autonomic phenomena. Subsequently, non‐paroxysmal neurological signs appear, such as cognitive impairment and movement disorders including choreoathetosis, dystonia, or ataxia. Mutations in the *ATP1A3* gene, encoding the α3 isoform of the Na^+^/K^+^ ATPase pump, are reported in ~85% of individuals.^1^, ^2^ Mutations in the same gene are also associated with other clinical syndromes.^3^, ^4^, ^5^, ^6^, ^7^

Disease course and progression in AHC is now a matter of debate. A non‐progressive course has been described in the past,^8^ whereas in a more recent case series, seven patients, aged 12 years and below, were reported to have experienced abrupt and irreversible regression along with significant acquired cortical and cerebellar atrophy.^9^ Slow and mild progression of non‐paroxysmal disability were recently observed in a multicentric cohort of 94 children and young adults.^10^

The only case of regression in adulthood described so far happened in concomitance with frequent episodes of status epilepticus, but few details regarding neurological function are given and the time course is unprecise.^11^

We report seven adult patients with AHC with detailed clinical history and long‐term follow‐up, adding data on the natural history of the disease and its phenotypic variability. Three of them presented acute regression, which we characterize with respect to clinical features, precipitating factors, genotype, and treatment.

## Methods

All patients and/or their parents/legal guardians gave informed consent/assent, including for video‐recordings. This study was approved by the local Ethics Committee. Clinical records of patients attending University College London Hospitals (UCLH), with a clinical diagnosis of AHC according to the Aicardi criteria,^12^ were reviewed. Video‐recordings of the neurological examination were acquired between May and September 2019.

Sequencing of the *ATP1A3* gene was performed in all individuals with the identification of a pathogenic *ATP1A3* mutation in six out of seven. One patient had whole‐exome sequencing, which did not identify any *ATP1A3* or other gene pathogenic variants.

## Results

We describe seven adult patients (six female, one male; age range, 25–42 years), diagnosed with AHC and followed‐up at our center for a median of 16 years (range, 7–24 years). The disease course for each individual is detailed in Table 1 and Video 1. During follow‐up, 3 of 7 patients experienced an episode of abrupt neurological deterioration in adulthood (age range at onset of deterioration, 19–34 years), following a febrile illness that triggered an extremely prolonged quadriplegic episode (n = 1), status epilepticus (SE) (n = 1), or both (n = 1). These adults went from being able to walk independently to being wheelchair‐bound, and they went from being mildly dysarthric to almost full loss of language function. One of them developed severe spastic tetraparesis with joint contractures and severe axial hypotonia with impaired head control (Video 1, patient 2). All three patients required percutaneous endoscopic gastrostomy (PEG) insertion because of severe dysphagia. One of the three died at age 29 years likely because of pneumonia (as stated on the death certificate), 10 years after the episode of acute neurological deterioration: she had had a cough and fever and post mortem computed tomography (CT) findings showed bilateral pulmonary consolidation. These patients with a history of severe regression carried three different mutations, S137F, S811P, and E815K. They were all on flunarizine as part of their regular treatment and had had no recent interruption in its use.

**TABLE 1:** Clinical features, diagnostic findings and treatment

Patient N	Age, gender	*ATP1A3* mutation	Age of onset (mo)	Symptoms at onset	Paroxysmal features	Non‐ paroxysmal features	Psychiatric features	Other	Baseline^a^ GMFCS score^23^	Last GMFCS score^23^	Intellectual disability	History of epilepsy and SE	History of acute regression	Treatment	EEG (age in yr)	MRI (age in yr)
1	42, F	c.2839G > A; p.G947R	3	Paroxysmal nystagmus and hemiplegic episodes, hypotonia	Hemiplegic episodes, paroxysmal dystonic attacks, myoclonus, headaches	Ataxia, hypotonia, chorea, dystonia, mild spastic paraparesis, dysarthria	Visual and auditory hallucinations, onset in her 30s	Scoliosis	2	2	Mild^b^	No	No	BAC	EEG (2, 18, 23, 30, 32) VT (34) some irregular theta activity	‐Normal (18) ‐Mild cortical and vermis atrophy (34)
2	34, F	c.2431 T > C; p.S811P	1	Paroxysmal eye fluttering, hypotonia	Hemiplegic and quadriplegic episodes, headaches, painful muscle spasms	Hypotonia, spastic tetraparesis, dysarthria	Depression		2	5	Moderate–severe^b^	Yes, last GTC sz age 25 yr	Yes (23 yr) after prolonged quadriplegic episode triggered by febrile illness	PHT, TPM, FLN, BAC	EEG (1) VT (26, 31) abnormal bursts of sharpened slow	‐Normal (23, 23.5, 24) ‐Cerebellar atrophy, vermis and superior hemispheres predominant (25)
3	27, M	c.2214A > C; p.S772R	12	Paroxysmal nystagmus and quadriplegic episodes, hypotonia	Hemiplegic episodes, paroxysmal dystonic attacks, headaches	Ataxia, dysarthria	No	Fatigue	1	1	Mild^b^	Yes, SE age 15 yr	No	TPM, FLN, PZT	EEG (11, 16) normal	Normal (19)
4	25, F	c.2839G > A; p.G947R	3	Dystonic attacks, hypotonia	Hemiplegic episodes, paroxysmal dystonic attacks, headaches	Ataxia, mild dysarthria	No	Fatigue	1	1	Mild^b^	No	No	No	EEG (1) normal	Normal (1)
5	37, F	None	4	Hemiplegic episodes, hypotonia	Hemiplegic and quadriplegic episodes, painful muscle spasms	Mild ataxia, ligamentous laxity	Depression, episodes of persecutory delusions with aggression	Fatigue	1	1	Mild	No	No	ZNS, FLN, PZT	EEG (9, 10, 11, 21, 24) VT (25) non specific theta abnormalities	Cerebellar atrophy (19, 24)
6	29, F (deceased)	c.2443G > A; p.E815K	2	Dystonic attacks, hypotonia	Hemiplegic episodes, paroxysmal dystonic attacks, myoclonus, headaches	Dystonia, choreoathetosis	No		4	5	Moderate–severe^b^	Yes, focal sz recorded during sleep age 24 yr	Yes (19 yr) after febrile illness	LCS, FLN, CLB	EEG (2, 3) normal VT (24) Focal spikes	Normal (10)
7	34, F	c.410C > T; p.S137F	1	Paroxysmal eye fluttering, hypotonia	Hemiplegic episodes, paroxysmal dystonic attacks, headaches, autonomic spells	Ataxia, dystonia	No	Fatigue	1	3	Moderate^b^	Yes, SE age 34	Yes (34 yr) after febrile illness and SE	LEV, FLN, PZT, CBZ	VT (23) diffuse slowing of background and generalized spike and slow waves (frontal dominant)	Left hippocampal sclerosis (5, 23)

^a^ Baseline motor performance as observed in the first encounter.

^b^ Formal neuropsychometry assessment was not available, intellectual disability severity was based on adaptive behavior according to the ICD‐10 classification.

Abbreviations: GMFCS, gross motor function classification system; SE, status epilepticus; VT, video‐telemetry; GTC sz, generalized tonic–clonic seizure; BAC, baclofen; PHT, phenytoin; TPM, topiramate; FLN, flunarizine; PZT, pizotifen; ZNS, zonisamide; LCS, lacosamide; CBZ, carbamazepine; CLB, clobazam.; LEV, levetiracetam; yr, yearrs.

**Video 1:** **Patient 1**. At rest, she shows mild dystonic features, more prominent on the right side and a few generalised jerky movements. Her speech is mildly dysarthric. With arms outstretched, dystonia of upper limbs becomes more evident. At the finger‐to‐nose test, there is some dysmetria on the right. Fine movements are impaired, more on the right, with great asymmetry. There is no true bradykinesia but some clumsiness bilaterally. Handwriting is legible but clearly dystonic. When walking, there is in‐turning of the feet, mainly on the right and abnormal posturing of both arms, flexed and with hands in a clenched fist. Posture is impaired, with mild antecollis. **Patient 2**. She is wheelchair‐bound with permanent right‐sided hemiplegia. She has also bilateral foot in‐turning with reduced articular mobility. When repeatedly stimulated, she can keep eye contact. Reaching movements are possible with the left arm. Head control is impaired, with limited ability to follow an object and sudden head drops. She has striatal toes and extension at the plantar reflex. **Patient 3**. When seated, he has mild truncal deviation towards the left. With arms outstretched, mild dystonia of the upper limbs becomes more prominent. There is some clumsiness during prono‐supination of the arms, along with mobile dystonia. A slight bilateral dysmetria is seen at the finger‐to‐nose test. At finger tapping there is no true bradykinesia, but the fluidity of the movement is bilaterally reduced due to the dystonia. In this segment of the video it is also possible to notice a reduced blink rate and a mild antecollis. Handwriting is barely intelligible, and severe dystonic posturing of the right hand while holding the pen is shown. While walking, hands are both held in clenched fist, with some involuntary proximal jerky movement of the arms. There is mild antecollis. He complains about fatigue, however he can walk independently for few meters. **Patient 4**. She is examined at the end of a prolonged right hemiplegic episode which evolved into paroxysmal dystonic attack. The right arm is still flexed, held in front of the body with the hand raised upwards. The rest of the examination is typical of her interictal condition. Her speech is clearly dysarthric. When asked to outstretch her arms and pronate/supinate the hands, she shows a bilateral, distal mobile dystonia with overflow phenomenon. No clear dysmetria at the finger‐to‐nose test, while fine hands movements are severely impaired on the right, due to the dystonia. However, handwriting is clear. Gait is independent with mild antecollis and some jerky movements of the left upper limb.

The remaining four patients developed other new symptoms in adulthood: fatigue (n = 4), psychiatric symptoms (n = 2), painful muscle spasms (n = 2), and scoliosis (n = 1). In particular, one individual showed significant deterioration of a pre‐existing movement disorder, with new onset of myoclonus (Video 1, segment 5); four individuals started to show signs of fatigue, becoming dependent on a wheelchair for short/medium distances, and two individuals started to complain of painful muscle spasms, which partially responded to baclofen. With respect to psychiatric issues, one patient experienced recurrent hallucinatory symptoms with aggression and the other had recurrent episodes of persecutory delusions on a background of low mood. To date, these symptoms have been relatively stable without the need for psychiatric treatment. One patient is awaiting psychiatric assessment.

## Discussion

Our cohort suggests that individuals with AHC may experience either abrupt or slowly progressive worsening of their neurological function and for most adults the hemiplegic episodes may no longer be the main clinical issue.

A previous longitudinal characterization of large AHC cohorts suggested a generally non‐progressive course of AHC,^8^ but adult patients represented a small proportion of the group (14/157). Review of the data in that report shows that 5 of 14 adults developed fine motor abnormalities after infancy (three in childhood, one in adolescence, and one in adulthood), 3 of 14 developed chorea and/or dystonia after infancy (two in late childhood, one in adulthood) and 2 of 14 developed new‐onset hypotonia and gross motor abnormalities after the age of 24 years. More recently, Uchitel et al^10^ described a large cohort of 94 children and young adults that they studied retrospectively and partly prospectively with a linear mixed effects model and observed a mild decline in non‐paroxysmal disability. Their methodological approach allowed to detect subtle degrees of progression and their cohort included 7 of 42 adults (age range, 20–43 years; mean follow‐up, 3.4 years) prospectively evaluated, and 23 of 52 adults in the retrospective analysis (19–39 years, mean follow up 9.9 years). In this cohort, a “catastrophic” regression is described in one subject, who did not have an *ATP1A3* mutation.

Other authors have already noted that movement disorders and spasticity can appear at a later stage,^13^ but, to the best of our knowledge, this is the first report of fatigue and muscle spasms in adults with AHC. Although muscle spasms could be interpreted as painful paroxysmal dystonic attacks, their predominance at night and their response to baclofen suggest they may be associated with chronic spasticity (as in multiple sclerosis).^14^ The same parallel with multiple sclerosis can be proposed for fatigue, the most common disorder where this symptom presents along with chronic spasticity. Fatigue could be also associated with the fear of exercise‐induced paroxysmal events.

In terms of psychiatric features, in a recent publication, 20/25 individuals with AHC had a neuropsychiatric diagnosis including attention deficit/hyperactivity disorder (ADHD), disruptive behavior, and mood disorders,^15^ but only three individuals were adults. Additionally, two individuals with a severe AHC phenotype and childhood‐onset schizophrenia have been reported.^16^ Milder adult‐onset psychiatric features have been described in two adult individuals with difficulties in social integration.^17^ It is not clear whether these psychiatric manifestations are independent comorbidities or if they are a new feature directly linked to the genetic mutation. We cannot exclude that psychiatric disorders in adults with AHC might be under‐reported.

Of our seven patients, three experienced an abrupt neurological regression in adulthood, following a febrile illness that triggered either or both an extremely prolonged quadriplegic episode and status epilepticus. There are at least 17 reports of abrupt neurological regression,^11^, ^18^, ^19^ although these were not always fully characterized (Table 2). Median age at regression was 6.5 years and only one patient was an adult.^11^ Of the 10 patients with available genetic data, six had the E815K mutation, which was proposed as a risk factor for acute regression. It was also observed that only one patient was on flunarizine, whereas the others had either recently discontinued it or never used it. In all these reported patients, catastrophic events were preceded by status or very frequent seizures. In our cohort, one of the three adult patients who experienced an acute regression had the E815K variant, whereas the others did not, and all were on flunarizine; regression was triggered by prolonged quadriplegia and fever and not only by seizure activity. Notably, none of the 13 patients (10 previously reported and three from our series) with a history of acute regression had the most common (D801N) recurrent mutation.

**TABLE 2:** Clinical and genetic features of patients with acute clinical regression

ID	Mutation	Age at regression (y)	Language regression	Motor function impairment	Precipitating events	Flunarizine treatment with respect to onset of clinical regression	References
Sasaki‐1	p.E815K	5	Words → absent	Stand → bed	SE, fever^a^	Discontinued 2 years before	17
Sasaki‐2	p.E815K	10	Absent	Bed	SE	Discontinued same year	17
Sasaki‐3	p.E815K	6	Sentence → words	Sit → bed	SE, fever^a^	Discontinued 1 year before	17
Sasaki‐4	p.E815K	7	Words	Stand → bed	SE	Discontinued 1 year before	17
Sasaki‐5	p.E815K	4	Words → absent	Stand → bed	SE	Discontinued 1 year before	17
Sasaki‐6	p.G755S	12	Words → absent	Stand → bed	SE	Ongoing treatment	17
Sasaki‐7	p.815 K	3	Words → absent	Sit → bed	SE	Never used	17
Uchitel‐21	Not identified	8	Sentence	Residual hemiparesis	SE	Never used	19
Uchitel‐32	p.V589F	2	–	Walk → bed	SE	Never used	19
Ito	p.G755S	38	–	Walk → bed	SE	Discontinued 13 years before	20
#2	p.S811P	23	Sentence → words	Walk → bed	Fever followed by quadriplegia	Ongoing treatment	Current study
#6	p.E815K	19	Words → absent	Crawl → bed	Fever followed by SE/quadriplegia^b^	Ongoing treatment	Current study
#7	p.S137F	34	Sentence → words	Walk → sit	Fever followed by SE/quadriplegia	Ongoing treatment	Current study

^a^ Information on whether fever preceded SE was not provided by the authors.

^b^ Difficult to distinguish between the two with the data reported, more detailed information available in the Supplementary data.

Abbreviations: Bed, bedridden; sit, sit without support; stand, stand with support; walk, walk unassisted; SE, status epilepticus. For the seven patients reported in Panagiotakaki et al^17^ the only information provided is that the regression was typically triggered by a “stressful event”.

In addition, there are 11 reports^8^, ^20^ of sudden unexpected death, an outcome that has recently been grouped together with regression as “catastrophic events in AHC.”^20^ The cause of these events and in particular the role of paroxysmal events and fever remain elusive. In 8 of 11 patients with sudden death, there were preceding prolonged paroxysmal episodes, either seizures or quadriplegic episodes.^8^, ^20^ Conversely, in a large clinical cohort, the previous degree of fixed disability emerged as the only significant risk factor for mortality, whereas the severity of paroxysmal events was not associated with early death.^8^ Episodes of abrupt onset or worsening of neurological signs triggered by fever or other stressors have been reported in other *ATP1A3*‐related conditions such as rapid‐onset dystonia parkinsonism^21^ and relapsing encephalopathy with cerebellar ataxia,^22^ where paroxysmal events are not a feature. This suggests that paroxysmal events and catastrophic events may be provoked by the same trigger, but are not causally linked.

Compared with the existing literature,^8,12,13^ we add here further characterization of the AHC course in adulthood and new information on genotype–phenotype correlation: we did not find an association between acute regression and mutation type or lack of flunarizine treatment as previously reported,^10,18,19^ although we acknowledge the small sample size of our cohort. In conclusion, our case series of patients with long follow‐up in adulthood provides useful insights on the natural history of AHC. In the long term, most patients have reduced frequency of paroxysmal events. However, adult patients may present with new onset of fatigue, muscle spasms, psychotic symptoms, and aggravation of movement disorders, all of which have a detrimental impact in their quality of life and level of independence. Finally, a sudden and irreversible deterioration of their condition, mainly with pyramidal and bulbar signs, may occur not only after status epilepticus, but also after prolonged non‐epileptic spells, which should, therefore, be promptly treated. Our report expands the spectrum of clinical and genetic factors associated with neurological regression in AHC. Understanding the evolution of disease in adulthood and its associated pathophysiology will help to design targeted interventions.

## Author Roles

(1) Research Project: A. Conception, B. Organization, C. Execution; (2) Statistical Analysis: A. Design, B. Execution, C. Review and Critique; (3) Manuscript Preparation: A. Writing of the First Draft, B. Review and Critique.

M.P.: 1A, 1B, 1C, 3A

J.P.: 1C, 3B

G.D.L.: 1C, 3B

S.D.A.: 1B, 3B

K.S.: 1B, 3B

S.Z.: 1B, 3B

D.J.J: 1B, 3B

D.B.: 1A, 3B

S.M.S.: 1A, 3B

S.B.: 1A, 3B

## Disclosures

### Ethical Compliance Statement

We confirm that we have read the Journal's position on issues involved in ethical publication and affirm that this work is consistent with those guidelines. This study was approved by the UCLH Ethics Committee. Patients have signed consent for video acquisition and publication.

### Funding Sources and Conflicts of Interest

The authors report no sources of funding and no conflicts of interest.

### Financial Disclosures for the Previous 12 Months

J.P., S.D., S.B., and S.M.S. are supported by the Epilepsy Society. S.B. is supported by the Muir Maxwell Trust. Part of this work was undertaken at University College London Hospitals, which received a proportion of funding from the National Institute for Health Research (NIHR) Biomedical Research Centres funding scheme. K.S. is supported by a Wellcome Trust Strategic Award (WT104033AIA).

## Supporting information

Summary of patients clinical history and investigations.
