# Supplementary material for: Non‐Stationary Outcome of Alternating Hemiplegia of Childhood into Adulthood
Source: Mov Disord Clin Pract. 2021 Dec 29;9(2):206–11. doi: 10.1002/mdc3.13388 (PMC8810436; doi:10.1002/mdc3.13388)
Supplement: Supplementary file 1 — Summary of patients clinical history and investigations. [file MDC3-9-206-s001.docx]

**Supplementary data**

***Patient 1***

Patient 1 is a 42 year-old lady with symptom onset at the age of 3 months with ‘jerky’ eye movements followed by transient hemi-body floppiness. She was also hypotonic and started walking at 3 years of age. From the age of 5 years, the paroxysmal episodes became mostly dystonic, triggered by anxiety and excitement, and resolved upon sleep. Since puberty, she has started suffering from migraines with visual aura; around the same period the frequency of the dystonic spells reduced from weekly to around monthly, as they are now. Currently they last less than two minutes and are associated with pallor and drooling. In terms of non-paroxysmal movement disorder, she presents features of chorea and dystonia with superimposed myoclonus; moreover, she is ataxic, dysarthric and mildly spastic in her lower limbs (video 1, segment 1).

Around age 35, she started to display episodes visual and auditory hallucinations associated with psychomotor agitation and sometimes aggressiveness, with a current monthly frequency. She is on treatment with baclofen 10mg/die. She tried flunarizine in the past but it was discontinued for inefficacy. She has mild intellectual disability. She attended special need school and college and she is now living in a residential home with 24/7 care but has relatively good independence. MRI was normal at the age of 18 years and subsequently showed mild cortical and vermian atrophy age 24. EEG throughout her life (2, 18, 23, 30, 32, 35 years) have always been normal, other than non-specific slow activities.

***Patient 2***

Patient 2 is a 34-year-old lady with symptom onset in the first month of life, when she started to display paroxysmal eye fluttering, followed at 2 months by episodes characterised by staring, pallor and generalized stiffness. ‘Classical’ hemiplegic spells followed at 9 months of age, lasting around two hours and presenting in clusters lasting four to five days, once or twice per month. Since the age of 4, she has suffered from migraine which would frequently precede the hemiplegic episodes. At the age of 10, after a reduction of the phenytoin dose, she experienced two focal to bilateral tonic-clonic seizures. She has not presented other similar episodes since.

At the age of 23, after a febrile illness, she presented a two-week episode of quadriplegia, associated with divergent strabismus and pendular nystagmus, anarthria and dysphagia; the lower limbs were more affected than the upper ones and the right side was more affected than the left. After this episode, she developed fixed spastic tetraparesis with relatively retained function in her left arm and became wheelchair-bound. In the last few years, her neurologic condition has further deteriorated, with worsening of her dysarthria, swallowing dysfunction for which she required PEG insertion, and progression of truncal hypotonia with fluctuant loss of head control (video 1, segment 2). In terms of paroxysmal events, she is currently experiencing mainly quadriplegic events, approximately twice a month.

The MRI at the age of 26 years old showed slight progression of cerebellar atrophy, involving mainly the vermis and the superior cerebellar hemispheres, compared to the previous imaging done at the time of the acute regression, which was normal.

***Patient 3***

Patient 3 is a 26 year-old gentleman. Hypotonia was noticed around the age of 8 months. At 12 months, he started to present episodes of generalised rigidity, apnoea, strabismus and perioral cyanosis. Subsequently, episodes of more clearly lateralised floppiness appeared, triggered by exercise or cold and relieved by sleep, which led to the diagnosis of AHC. Since the age of 8, he started experiencing episodes of paroxysmal dystonia of the arm and the leg, more commonly on the left side, which lasted usually for a few hours, associated with pallor, paroxysmal strabismus and headache, the latter occurring also in isolation. At 15 years of age, he was admitted to ICU for ventilatory support after a prolonged generalised tonic-clonic seizure (>1h), and required general anaesthesia. In the last years, he has experienced only hemiplegic episodes with a yearly frequency. He is ataxic and dysarthric (video 1, segment 3). However, his main concern has become an increasingly invalidating fatigue and now he is not able to walk for more than 100m without support. He has mild intellectual disability, he attended special need school and college and now lives in a residential home with support. He is on treatment with topiramate, flunarizine and pizotifen. His brain MRI, performed at age 19 years, was unremarkable.

***Patient 4***

Patient 4 is a 24 year-old lady. First concerns arose at the age of 4 months, when she started experiencing episodes of head and eye deviation, with fully retained consciousness, lasting up to several hours. In the following months, some nystagmoid eye movements were noticed. Since she was 1 year-old she started to have ‘classical’ alternating hemiplegic episodes which could evolve to dystonic posturing of the arms. The most severe episodes would progressively involve also the lower limbs, first unilaterally and then bilaterally. Headache and sometimes severe wrist pain could occur ‘in waves’. Sometimes, also brainstem signs with drooling, difficulty in breathing and swallowing would appear. The episodes are typically triggered by fear or excitement and resolve upon sleep. Throughout the years, the semiology of her spells has remained approximately constant, but their duration and frequency have very slowly progressed, and she is now having episodes of variable intensity about 70% of the days. Interictally, she is mildly ataxic and the distance she is able to walk without support has reduced and is now 500 meters at most (video 1, segment 5). She attended mainstream school with low grades and is currently attending a professional course; she is considered to have mild intellectual disability, but she has not received formal neuropsychological testing. EEG and MRI performed in her first year of life were normal.

***Patient 5***

Patient 5 is a 37 years old lady who was born at 31 weeks of gestational age but presented no major complications of prematurity. At 4 months she was noticed to drag the left side of the body while crawling and this could be followed by transient generalised floppiness and cyanosis. Her psychomotor development was only slightly delayed, with stable independent walking at 2 years. Around this time, her paroxysmal episodes, occurring four or five times per week, changed: they were described as ‘drop attacks’ involving the head and/or the trunk, followed by dysarthria and dysphagia, pallor, mydriasis and, sometimes, twitching movements of the arms and leg on alternating sides could follow. She required several hospital admissions since these attacks could be extremely prolonged; she would unable to speak and swallow for days and required admission for intravenous hydration. The attacks were often triggered by cold temperatures and excessive tiredness and resolved upon sleep. These episodes were initially thought to be epileptic in nature and she was prescribed with multiple combinations of antiseizure medications (including phenytoin and primidone), ketogenic diet and high doses of corticosteroids, with no improvement. When she was 11-year-old, a diagnosis of AHC was made, considering the disease course, clinical observations of the attacks and the normal EEG during them. Therefore, all antiseizure medications were stopped and flunarizine was started, with an improvement of the duration and severity of the attacks, which have not required hospital admission ever since and are now frequent (20-30/month) but short-lasting. Since the age of 22, she has started to complain of painful muscle spasms affecting her back, wrists and ankles, lasting for hours, which are more frequent at night, keeping her awake, and partially responded to oral baclofen. At the same time, she started complaining of fatigue and began to use a wheelchair for longer distances (video 1, segment 4). She attended mainstream school and went to college. She currently lives with her parents but is relatively independent. Her last WISC scale performed at 15 years revealed a total IQ of 65; verbal skills were a relatively strength in her profile. EEG throughout life (9, 10, 11, 21, 24, 25 years) have never shown epileptiform abnormalities while MRI showed cerebellar atrophy stable between the age of 19 and 24 years.

***Patient 6***

Patient 6 passed away at the age of 29 years. As a newborn, she was admitted for poor feeding and lethargy and presented an episode of hypertonus and cyanosis. At 6 weeks of age, she was noted to ‘shake’ the right leg in a stereotyped way along with poor visual development, wandering eye movements, poor swallowing and hypotonia. At the age of 18 months, she started to experience hemiplegic episodes with mixed flaccid and tonic features, which could last from hours to days; she was started on flunarizine, with some benefit. Her psychomotor development was slow but constant; she acquired standing position by the age of 3 years. She was also having episodes of staring and generalised atonia, along with myoclonic jerks. The EEGs done at the time have always been deemed normal. Various antiseizure medications including phenytoin, clobazam and levetiracetam were administered with no success; the same applied for vagal nerve stimulation. Since the age of 10 she started to use a wheelchair for a progressively deteriorating movement disorder characterised by generalized dystonia with choreoathetoid features. Her motor and verbal skills declined abruptly at the age of 19 after an episode of high fever, followed by a prolonged episode with mixing features of dystonia, hypotonia and clonic jerks, which have been interpreted as seizures; her awareness during the episode was unclear. After the episode, she became unable to talk and to support her head. She required PEG insertion because of severe dysphagia and also her manual skills deteriorated. A 24h-EEG when she was 24 years old recorded two focal onset seizures (with dystonic posturing and head deviation) during sleep and many non-epileptic events labelled by the family as seizures (limbs twitching, eye-rolling, throaty sounds). Interictal EEG showed frontal right spikes and polyspikes on a generalised slow background. At the age of 29, she passed away suddenly in the context of a chest infection. Six weeks before the death, her mother noticed for the first time an episode of prolonged apnoea (lasting at least 30s) with paleness. A recent 72h-ECG recording had showed no abnormality in the heart rhythm.

***Patient 7***

Patient 7 is a 34-year-old lady. At three weeks of age, she showed episodes of eye flickering while she was bathing. At three months, she started to have dystonic episodes characterised by eye and head turning, arm posturing and back arching, lasting several hours to days, requiring sometimes nasogastric feeding. She then developed classical alternating hemiplegia episodes, lasting from minutes to hours. Her psychomotor development was very slow but steady and at 7 years she was able to walk and talk in short phrases. She had her first generalised tonic-clonic seizure when she was 12, and these subsequently continued yearly. At the age of 22 she started to display episodes of autonomic dysfunction with paleness and then cyanosis, associated with reduced consciousness, lasting around 1 minute. The following year, she performed a 67h-EEG that recorded only hemiplegic spells associated with no EEG change, over a diffusely slow background and occasional generalized spike wave discharges. Brain MRI showed left hippocampal sclerosis. A cardiac loop recorder was implanted, and showed three episodes of asystole over four months, the longer lasting 5 seconds. It was therefore decided to implant a dual chamber pacemaker. In terms of motor function, at the age of 32 years she was able to walk short distances with an ataxic gait, use a spoon and play table games. She was able to express verbally with short sentences. She had dystonic episodes triggered by walking, so she started to use a wheelchair for most of the time. In the last year she experienced several episodes of prolonged quadriplegia and SE, often after febrile illnesses, which required hospitalisation and in three instances intensive care and ventilation. A PEG had to be placed due to dysphagia and her motor and verbal skills have regressed. She is not able to walk anymore and also trunk control is now fluctuant. In terms of communication, she speaks a few words but becomes easily fatigued.
